# Clinical significance of cortical lesions in patients with multiple sclerosis: A neuropsychological and neuroimaging study

**DOI:** 10.1002/brb3.934

**Published:** 2018-02-14

**Authors:** Futoshi Matsushita, Hirotaka Kida, Ken‐ichi Tabei, Chizuru Nakano, Keita Matsuura, Yuichiro Ii, Ryogen Sasaki, Akira Taniguchi, Yugo Narita, Masayuki Maeda, Masayuki Satoh, Hidekazu Tomimoto

**Affiliations:** ^1^ Department of Dementia Prevention and Therapeutics Mie University Mie Japan; ^2^ Department of Occupational Therapy Morinomiya University Osaka Japan; ^3^ Department of Neurology Mie University Mie Japan; ^4^ Department of Radiology Mie University Mie Japan

**Keywords:** cognitive impairment, cortical lesions, magnetic resonance imaging, multiple sclerosis, neuropsychological tests

## Abstract

**Introduction:**

This study aims to investigate the association between the presence and frequency of cortical lesions (CLs), and the clinical and psychological features of multiple sclerosis (MS).

**Methods:**

A total of 19 patients with MS were examined using double inversion recovery (DIR) sequences with 3T magnetic resonance imaging (MRI) and classified into two groups: CL and non‐CL. In‐house software was used to quantitatively determine the atrophy of each brain region. Activities of daily living (ADL) were estimated using the Kurtzke Expanded Disability Status Scale (EDSS). Cognitive function was assessed using the following tests: Mini‐Mental State Examination (MMSE), Trail Making Test (TMT), and Paced Auditory Serial Addition Task (PASAT). *Z*‐scores were used to assess significant differences in the neuropsychological test outcomes between the groups.

**Results:**

Six of 19 patients had subcortical and deep WM lesions (non‐CL group; diagnosed with relapsing–remitting MS). Thirteen of 19 patients had both subcortical and cortical lesions (CL group; 9—relapsing–remitting MS; 4—primary/secondary progressive MS). There were no significant differences in age, education, and disease duration, but EDSS scores were significantly higher in the CL group compared to the non‐CL group. There were no significant differences in gray and white matter volume between the CL and the non‐CL groups, but the white matter lesion volume was significantly higher in the CL group compared to the non‐CL group. Neuropsychological tests showed significant performance worsening in the CL group as compared to the standard values for healthy individuals in their age group, especially in the TMT data.

**Conclusions:**

Progressive MS, which was associated with decreased physical functioning, ADL, and cognitive impairment, was found in patients in the CL group.

## 1 INTRODUCTION

Multiple sclerosis (MS) is a typical inflammatory demyelinating disease of the central nervous system. In the past, MS was considered a disease that primarily affects white matter (WM). However, demyelinating lesions have also been observed in cortical and central gray matter (GM) (Filippi & Rocca, 2010). Although cortical demyelination and cortical lesions (CLs) are a characteristic feature of progressive multiple sclerosis, that is primary progressive MS (PPMS) and secondary progressive MS (SPMS), WM lesions are mainly present in acute and relapsing–remitting MS (RRMS) (Mahad Don & Bruce, 2015). Pathologically, B‐cell follicle‐like structures are detected in the inflamed meninges of some patients with MS, which correlate with increased subpial demyelination and cortical atrophy (Magliozzi et al., 2007). CLs particularly occur when there is microglial activation in the lymph follicle structures (Howell et al., 2011). These ectopic lymph follicle‐like structures contain abundant B cells and plasma cells and cause demyelination in the cerebral cortex adjacent to the lymph follicle (Magliozzi et al., 2007).

Although autopsy of brains from patients with MS showed CLs, which were primarily associated with progressive MS (PPMS and SPMS) and rarely with RRMS (Kutzelnigg et al., 2005), the advent of the MRI double inversion recovery (DIR) method enabled a high rate of CL detection (Filippi & Rocca, 2011). The incidence of CLs is now known to be higher than previously expected, irrespective of the stage of disease (Lucchinetti et al., 2011). Indeed, CLs have been detected in 74.2% of SPMS and 64.4% of RRMS cases using 3D DIR imaging (M. Calabrese et al., 2010). Further, 3D DIR imaging allows the quantitative investigation of pathological features of MS with CLs, with a higher diagnostic accuracy than imaging using 3D fluid‐attenuated inversion recovery (FLAIR) (Seewann et al., 2012).

In MS, cognitive impairment is found in 40%–65% of all patients (Rao, Leo, Bernardin, & Unverzagt, 1991). Such cognitive impairment primarily includes attention impairment, the reduced ability to process information, executive function disorder, and disturbance of memory (Benedict & Zivadinov, 2011). Overall cognitive and speech functions, however, are relatively maintained (Bobholz & Rao, 2003). Cognitive impairment in MS has been attributed to brain atrophy (Lanz, Hahn, & Hildebrandt, 2007), total lesion load (Benedict et al., 2004; Sanchez, Nieto, Barroso, Martin, & Hernandez, 2008), and the presence of CLs, which may be associated with attention impairment and working memory deficits (Rinaldi et al., 2010). In addition to cognitive impairment, CLs have been correlated to physical disability with an increased Kurtzke Expanded Disability Status Scale (EDSS) score (Mike et al., 2011; Nelson et al., 2011).

However, it is not clear how various pathological features, such as CLs, subcortical WM lesions, and brain atrophy, differentially affect cognitive function in different types of MS (M. Calabrese et al., 2015; Rocca et al., 2015; Steenwijk et al., 2015; Yaldizli et al., 2016). No study has comprehensively described the association between CLs, attention impairment, working memory deficits, and a decrease in EDSS score. Thus, the purpose of this study was to investigate the association between the presence and frequency of CLs, and the clinical and psychological features of MS, in a cohort of patients with MS. Hence, we performed 3T MRI with a DIR sequence and various neuropsychological tests and assessed the EDSS scores of 19 patients, who underwent examination at the outpatient services of the Department of Neurology of Mie University Hospital, Japan.

## 2 MATERIALS AND METHODS

### 2.1 Study population

The study population comprised 19 patients with MS (8 men and 11 women; mean age, 44.2 ± 9.9 years) who were examined at Mie University Hospital between July 2011 and October 2014. This study was approved by the ethical review board of Mie University Hospital, and with the Helsinki Declaration of 1975, as revised in 2008. Written informed consent was received from all participants, who were provided a written explanation of the study prior to participation.

### 2.2 Clinical and neuropsychological assessment

Disease type, disease duration, and EDSS scores were then noted using the clinical data sheets completed by the doctors in charge of each participant. Cognitive function was examined using the Mini‐Mental State Examination (MMSE), Japanese Raven's Colored Progressive Matrices (RCPM), the Rivermead Behavioral Memory Test (RBMT), the Trail Making Test (TMT) versions A and B, word fluency (WF) tasks, visuospatial construction tasks, and the 1‐ and 2‐s versions of the Paced Auditory Serial Addition Task (PASAT). The academic history of each was confirmed when performing cognitive function testing.

### 2.3 MRI

Magnetic resonance imaging was performed using 3T‐MRI scanners (Achieva; Philips Health Care, Best, the Netherlands). 3D‐FLAIR and T1‐weighted images were obtained using the following parameters: for 3D FLAIR—repetition time (TR), 6,000 ms; echo time (TE), 310 ms; inversion time, 2,000 ms; field of view (FOV), 25 cm; matrix size, 480 × 256; and section thickness, 1.14 mm; for T1‐weighted scans—TR, 7.6 ms; TE, 3.6 ms; flip angle, 8°; FOV, 250 mm × 250 mm; in‐plane resolution, 1.04 × 1.04 mm; and slice thickness, 1 mm.

### 2.4 Image analysis

**Figure 1:**
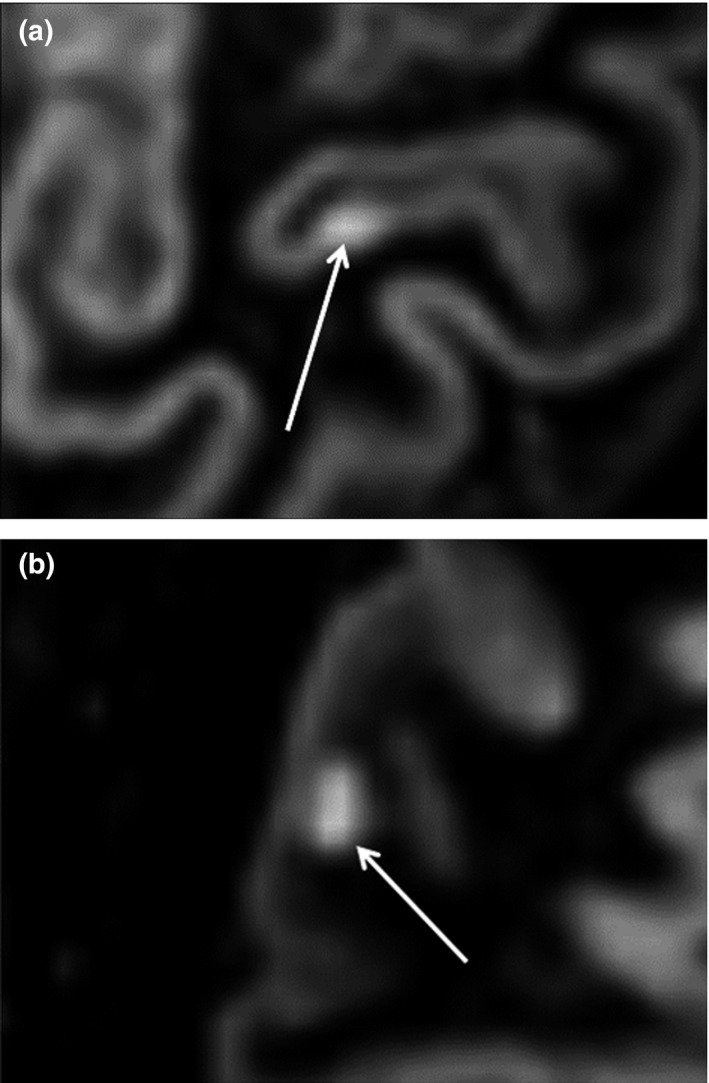
Representative 3‐T DIR images showing (a) a cortical lesion, and (b) a subcortical (white matter) lesion

Double inversion recovery axial, coronal, and sagittal section images from 3T MRI scans of the 19 participants were obtained and analyzed by two neurologists. Lesions affecting the cortical (gray matter) were judged as CLs. The participants were classified into either the CL or non‐CL group, based on the presence of absence of CLs, respectively (Figure 1). Tissue quantification was performed using in‐house software (Fused Software for Imaging Of Nervous system: FUSION) (Tabei, Kida, Hosoya, Satoh, & Tomimoto, 2017), which yielded an individualized volumetric profile of brain tissue. The obtained T1‐weighted and FLAIR images were imported from DICOM format files for processing. We used the lesion segmentation tool for lesion filling to increase the accuracy of segmentation. Lesion filling was applied to T1‐weighted images that were aligned with the lesion probability map. First, the T1‐weighted images were coregistered to FLAIR images for adaptive level preprocessing. Next, GM and WM segmentation was performed in T1‐weighted and FLAIR images using the Montreal Neurological Institute template as the reference probability map. The preprocessing function was based on SPM 8 (Wellcome Trust Centre for Neuroimaging, UCL). Next, second‐level tissue segmentation was performed to separate the WM lesions from the WM using a semi‐automated operation that extracted the pixels, which were within a predetermined value. The volume of WM lesions, which appeared as hyperintense areas on FLAIR images, was quantified. The GM and WM volumes and the ratios of cortical and WM volume to entire brain volume were also quantified.

### 2.5 Statistical analysis

Data analysis was performed using SPSS Version 22.0 (IBM) software. Differences in the age, EDSS score, TMT‐A, category WF task performance, drawing visuospatial construction task, 1‐s PASAT and 2‐s PASAT performance, cortical and WM volume between the CL and non‐CL groups were assessed using Student's *t* tests. The Mann–Whitney *U* test was used to investigate academic history, disease duration, and performance on the following neuropsychology tests: MMSE, RCPM score and time, standardized profile score (SPS), and screening scores (SS) in the RBMT, TMT‐B, letter WF task performance, copying visuospatial construction task. WM lesion volume between the CL and non‐CL groups was assessed using Welch's *t* test. The level of statistical significance was set at *p* < .05 for all statistical analyses.

Neuropsychological assessment outcomes were statistically significantly different between the CL and non‐CL groups were further analyzed using *z*‐scores to evaluate the deviation from the mean values for healthy individuals of their age group (Takeda et al., 2011). Briefly, the *z*‐scores were calculated for each participant as [(individual score–age group‐specific standard mean value)/standard deviation]. Thus, *z*‐scores ranked patients after inherently adjusting for any age differences between the groups. An abnormally low score was defined as being 1.5 standard deviations (SDs) or more below the normal value for each age group. The number of participants with a low *z*‐score was determined for each group.

## 3 RESULTS

A total of six of 19 patients had subcortical and deep WM lesions (non‐CL group), while 13 of 23 patients had additional cortical lesions (CL group). All six patients in the non‐CL group had RRMS. In the CL group, nine patients had RRMS, one patient had PPMS, and three patients had SPMS (Table 1). There were no significant differences in age, education, and disease duration between the CL and the non‐CL groups. The EDSS scores were significantly higher in the CL group (2.8 ± 1.8) than those in the non‐CL group (0.5 ± 0.8; *p =* .009) (Table 2).

**Table 1:** Clinical data of participants in the cortical lesion (CL) and non‐CL groups

Age (years)	Sex	Disease duration (years)	Disease type	EDSS	Education period (years)	Current disease modifying therapy
CL group						
44	M	8	RRMS	2.5	21	IFNβ
18	F	5	RRMS	0	12	—
46	F	11	RRMS	2	12	IFNβ
40	F	7	RRMS	0	16	IFNβ
56	M	23	RRMS	3	12	Corticosteroid, Fingolimod
53	M	22	RRMS	2.5	12	Fingolimod
43	F	23	RRMS	3	12	—
42	M	12	RRMS	3	12	IFNβ
37	M	4	RRMS	2.5	16	IFNβ, Corticosteroid
52	F	5	PPMS	3.5	12	IFNβ
43	F	25	SPMS	6	12	—
53	M	24	SPMS	6	12	Corticosteroid
33	F	11	SPMS	2	14	IFNβ
Non‐CL group						
44	F	9	RRMS	0	18	IFNβ
51	M	4	RRMS	2	12	IFNβ, Corticosteroid
40	F	27	RRMS	0	16	—
33	M	12	RRMS	0	12	IFNβ
58	F	12	RRMS	0	12	—
54	F	36	RRMS	1	16	—

EDSS, Kurtzke Expanded Disability Status Scale; IFNS, interferons; PPMS, primary progressive multiple sclerosis; RRMS, relapsing–remitting multiple sclerosis; SPMS, secondary progressive multiple sclerosis.

**Table 2:** Demographics, clinical and neuropsychological assessments of participants in the cortical lesions (CL) and non‐CL groups

	CL *n* = 13, 6 (M)	non‐CL *n* = 6, 3 (M)	*p*‐value
Demographics			
Age	43.1 (10.1)	46.7 (9.4)	.47
Education (years)	13.5 (2.7)	14.3 (2.7)	.39
Disease duration (years)	13.8 (8.2)	16.7 (12.2)	.51
EDSS			
Score	2.8 (1.8)	0.5 (0.8)	.009*
Neuropsychological assessments			
MMSE	27.9 (2.5)	28.8 (1.5)	.37
RCPM			
Score	31.0 (4.5)	34.0 (2.0)	.09
Time (s)	376.5 (324.3)	252.5 (79.8)	.86
RBMT			
SPS	19.5 (5.7)	22.5 (1.0)	.21
SS	9.2 (2.8)	10.8 (1.0)	.25
TMT			
A (s)	109.0 (66.5)	51.2 (40.7)	.068
B (s)	148 (99.9)	68 (29.7)	.009*
WF			
Category (/min)	17.1 (4.1)	20.3 (2.4)	.09
Letter (/min)	7.3 (1.7)	8.0 (2.0)	.31
Construction			
Copy	2.8 (0.4)	2.8 (0.4)	.75
Drawing	3.0 (0.0)	3.0 (0.0)	1
PASAT			
1 s	31.0 (19.0)	46.3 (9.6)	.081
2 s	49.6 (28.9)	67.5 (20.2)	.19

EDSS, Kurtzke Expanded Disability Status Scale; MMSE, Mental State Examination; PASAT, Paced Auditory Serial Addition Task; RBMT, Rivermead behavioral memory test; RCPM, Japanese Raven's Colored Progressive Matrices; TMT, Trail Making Test; WF, word fluency.

*p < 0.05.

There were no significant differences in the GM and WM volumes between the CL and the non‐CL groups. The GM volume to brain volume was 45.0 ± 6.0% in the CL group and 45.0 ± 5.4% in the non‐CL group (*p = *.99). Similarly, WM volume to brain volume was 32.7 ± 2.5% in the CL group and 34.5 ± 4.4% in the non‐CL group (*p *=* *.26). However, WM lesion volume was significantly higher in the CL group compared to the non‐CL group (15.4 ± 8.6 cc vs. 6.8 ± 3.1 cc, *p = *.005; Table 3).

**Table 3:** Gray matter (GM), white matter (WM), and WM lesion volumes of patients in the cortical lesion (CL) and non‐CL groups

	CL group (*n* = 13)	non‐CL group (*n* = 6)	*p*‐value
GM volume (%)	45.0 (6.0)	45.0 (5.4)	.99
WM volume (%)	32.7 (2.5)	34.5 (4.4)	.26
WM lesion volume (cc)	15.4 (8.6)	6.83 (3.1)	.005

With regard to cognitive function, there were no significant differences in the MMSE score, RCPM score, RCPM time, RBMT SPS, RBMT SS, TMT‐A, category WF task performance, letter WF task performance, visuospatial construction copying performance, visuospatial construction drawing performance and 1‐s PASAT, and 2‐s PASAT between the CL and non‐CL groups (Table 2). The TMT‐B values were significantly higher in the CL group than those in the non‐CL group (148.2 ± 99.9 s vs. 67.5 ± 29.7 s, *p = *.009). Thus, differences between the CL and non‐CL groups were significant in more difficult tests, that is the TMT‐B (Table 2).

The *z*‐score analysis of the TMT and PASAT values showed significant performance worsening in the CL group as indicated by a higher proportion of patients with abnormally low *z*‐scores (TMT‐A, TMT‐B, and PASAT 1 s: CL group 4/13 vs non‐CL group 0/6; PASAT 2 s: CL group 7/13 vs non‐CL group 1/6; Table 4).

**Table 4:** *Z*‐score analysis of clinical assessment for cognitive function and attention of patients in the cortical lesion (CL) and non‐CL groups

Task	Assessment	Patients (*n*) with *Z*‐score < −1.5 *SD*	
		CL group (*n* = 13)	non‐CL group (*n* = 6)
PASAT 2 s	% correct answer	7	1
PASAT 1 s	% correct answer	4	0
TMT Part A	Completion time	4	0
TMT Part B	Completion time	4	0

*Z*‐score = (Individual score‐age group‐specific standard mean value)/standard deviation.

PASAT, Paced Auditory Serial Addition Task; *SD*, standard deviation; TMT, Trail Making Test.

## 4 DISCUSSION

The results of this study can be summarized as follows: 1) CLs were observed in 13 of 19 patients with MS; 2) all patients with PPMS and SPMS had CLs; 3) there were no differences in the age, education, disease duration, and brain compartment volumes between the CL group and non‐CL group; 4) compared to the non‐CL group, the CL group had significantly larger WM lesion volumes and lower EDSS scores; 5) the CL group had significantly poorer results on neuropsychological testing, as indicated by the larger proportion of patients with attentional dysfunction, defined by *z*‐scores below 1.5 SDs.

A previous study reported that patients with MS presented with attentional impairment, reduced information processing ability, and cognitive impairment in addition to physical disability (Benedict & Zivadinov, 2011). The results of the current study corroborated with these previous results as the presence of CLs in MS was associated with ADL impairment and attentional dysfunction. Indeed, the CL group showed significantly lower EDSS scores, which are mainly used to evaluate motor function and walking ability, primarily based on neurological findings. Further, of various neuropsychological tests, the outcomes for the TMT‐B demonstrated abnormalities in the CL group. As these tests primarily evaluate attentional function, higher brain dysfunction in MS can be primarily attributed to attentional dysfunction (Rinaldi et al., 2010). Several previous studies (Harrison et al., 2015; Kolber et al., 2015; Papadopoulou et al., 2013; Sethi et al., 2016) have reported an association between CLs and cognitive and physical impairment in MS, in agreement with the present findings.

Volumetric analysis demonstrated that WM lesion volume was significantly greater in the CL group than in the non‐CL group, suggesting that progression WM lesions accompany the presence of CLs. This is corroborated by previous reports, which demonstrated that cognitive impairment in MS depends on the total number of lesions, rather than on brain atrophy (Benedict et al., 2004; Papadopoulou et al., 2013; Sanchez et al., 2008). Conversely, Amato et al. (2004, 2007) showed the presence of cortical atrophy in patients with MS who had cognitive impairment. The present study demonstrated that both cognitive impairment and WM lesions, but not gray and white matter atrophy, accompanied CLs. As cognitive impairment in MS is known to progress over 5–7 years (Deloire, Ruet, Hamel, Bonnet, & Brochet, 2010), it is possible that CLs influence cognitive function earlier than brain atrophy in patients with MS.

Between the CL group and non‐CL group, there were no differences in age, education, and disease duration. This signifies that, in MS, CLs develop regardless of age and disease duration. Magliozzi et al. (2007) analyzed autopsied brains of patients with SPMS and detected B‐cell follicle‐like structures in the inflamed meninges in more than half of the patients with SPMS. Like progressive MS, inflammatory cortical demyelination, consisting of microglial activation, neurodegeneration, and reduced oligodendrocytes, has also been observed early during the course of MS (Geurts & Barkhof, 2008; Lucchinetti et al., 2011). Indeed, an MR‐based study revealed both focal CLs and generalized GM abnormalities more frequently in SPMS than in RRMS (Yaldizli et al., 2016). Further, changes in extralesional GM were found to be more consistently associated with disability compared with CLs. Therefore, cognitive impairment in MS can be attributed to the pathologies in both cerebral GM and WM (P. Calabrese & Penner, 2007; Papadopoulou et al., 2013; Rocca et al., 2015; Steenwijk et al., 2015).

A few limitations in the present study must be noted. Given the single‐center cross‐sectional design of the current study, the sample size was relatively small. Larger sample sizes are required for complex multivariate statistical analyses to clarify whether the differences between groups are a consequence of the absence or presence of CLs or of the disease state of patients with MS. Further, as clinical progress of individual patients was not monitored, it was difficult to determine whether CLs and cognitive impairment appeared when RRMS transformed into progressive MS or whether progressive MS and RRMS were completely different entities. Previous studies demonstrated an increase in the correlation of CLs with the exacerbation of EDSS score in RRMS (M. Calabrese et al., 2010) and cognitive impairment in all MS types, including RRMS, SPMS, and PPMS (M. Calabrese, Agosta, et al., 2009; M. Calabrese, Rocca, et al., 2009; Roosendaal et al., 2009). Early‐stage cognitive impairment in MS, however, only served as an indicator predicting the exacerbation of EDSS score after 5–7 years (Deloire et al., 2010). Thus, the difference in CLs and cognitive function between RRMS and progressive MS needs to be examined in a longitudinal study. In addition, future studies using pathological and genetic analysis are needed to further clarify how clinical and genetic characteristics are related to specific pathologies in cortical lesions and cognitive impairment (Tauhid, Neema, Healy, Weiner, & Bakshi, 2014). Finally, we did not have access to the Brief Repeatable Battery of Neuropsychological Tests, which was developed for the efficient detection of cognitive impairment in MS. Nonetheless, the PASAT and TMT in the present study exhibited a reasonable detection power, despite the lack of significant differences noted in the scores for the MMSE, RCPM, RBMT, WF tasks tests.

## 5 CONCLUSION

In conclusion, 3T MRI using DIR sequences, and clinical and neuropsychological testing were used to comprehensively investigate the relationship between CLs, and cognitive and physical impairment in MS. CLs were more frequently observed in progressive MS and were associated with greater WM lesion volume, decreased physical functioning, ADL, and cognitive impairment, but not cortical atrophy. Larger, longitudinal studies are needed to further clarify the effect of CLs in cognitive function in various types of MS.

## CONFLICT OF INTERESTS

This study received no specific grant from any funding agency in the public, commercial, or not‐for‐profit sectors. Furthermore, none of the authors has any commercial or financial involvement in connection with this study that represents or appears to represent any conflict of interests.

## ETHICAL STANDARD

All patients provided written informed consent for examination before participating in the study, which was approved by the local ethics committee.
